# Healthcare needs and programmatic gaps in transition from pediatric to adult care of vertically transmitted HIV infected adolescents in India

**DOI:** 10.1371/journal.pone.0224490

**Published:** 2019-10-29

**Authors:** Archana Verma, Seema Sahay

**Affiliations:** Division of Social and Behavioral Research, Indian Council of Medical Research-National AIDS Research Institute (ICMR-NARI), Pune, India; University of Ghana College of Health Sciences, GHANA

## Abstract

Treatment transition for 'adolescents living with perinatally acquired HIV' (ALPH) from paediatric to adult care is not addressed adequately. This study explores the ALPH's health care needs and programmatic gaps in health systems for the care of ALPH in India. Forty-nine in-depth interviews were conducted with purposively selected primary and key stakeholders in India. Thematic analysis utilizing grounded theory was performed in QSR NUD*IST 6. Stakeholders explicitly recognized adolescent HIV to be a critical public health issue which requires a separate mandate in India. It was found that none of the health policies in India focus on adolescent age group; ALPH is therefore even more neglected population. No/partial HIV disclosure to ALPH is the first crisis for retention in care continuum and adherence to the treatment becomes sub-optimal. Unmet needs of transitioning from paediatric to adult care in existing settings was the major gap. Age-specific counselling guidelines and counselling skills among HCPs were found lacking where tailored counseling and capacity building of HCPs was an expectation. Need of holistic approach for adolescents led to consensus on establishing 'adolescent transition clinic' with a strict 'no' for 'standalone Adolescent HIV' clinics. School setting having peer-based counselling provision was recommended. Age disaggregated health data is required to inform the policymakers about adolescents’ specific needs for developing interventions. Situational analysis to identify and shape health priorities of adolescents is recommended.

## Introduction

An estimated 1.2 million premature deaths occur among adolescents, mostly from preventable or treatable causes [1,2]. Among 1.2 billion world’s adolescents, two million are living with HIV [3]. AIDS related deaths have tripled among adolescents since 2000, making HIV/ AIDS the second leading cause of adolescents' death [1,3].

With successful implementation of the ‘Prevention of Parent to Child Transmission’ (PPTCT) program and strong investment in HIV treatment, India witnesses an increased survival of children who acquired HIV perinatally. Children (>15 years) account for 6.54% of people living with HIV in India [4] who will soon add to the emerging generation of adolescents in future. But this subset of the population, is the less healthy subset of the adolescent population who face the prospect of having to take ART daily with optimum adherence for the rest of their lives [4]. **A**dolescent **L**iving with **P**erinatally acquired **H**IV (ALPH) are in double peril as they go through adolescence as well as HIV; facing the bodily changes of growing up as well as the health challenges on account of reduced immunity due to HIV infection [5]. Additionally, sustaining adherence for chronic disease/s is one of the major health threatening challenge among adolescent population living with chronic illnesses [6,7], and HIV is no exception [8]. However, HIV research studies and programs often either exclude adolescents or group them either with pediatric (below 15 years) or adult (above 15 years) population group respectively [9]. Management strategies for adults are replicated among adolescents [10,11]. There is a need to understand that it is a separate group in itself with its own requirements.

The World Health Organization (WHO) defines an adolescent as any individual between 10 and 19 years of age, but young adolescents, aged 10–14, are often invisible in discourse and data, falling between policies and programmes focused on “children” and “youth” respectively [12,13]. According to the National AIDS Control Programme (NACP-III): “*There is no provision of psychosocial support strategies in the government set up which is essential for the children who are infected and have to cope with sense of loss and insecurity*” [14]. Hence, there is a need to examine the existing gaps in health care settings and needs of health care providers (HCPs) to manage the ALPH in India. An understanding of health care needs and gaps in system would inform the program in identifying the strategies to improve ALPH care.

## Methods

### Theoretical framework

This study is an examination and an exposure of existing systemic processes that can influence health care and program experiences among ALPH. We have used critical realists paradigm [15,16] to conduct this study. We aimed to collect contextual data among HCPs to understand the needs of ALPH. Data from experiences of ALPH pertaining to health care was studied. We used Donabedian's health care quality framework [17] to identify themes for optimization of clinical processes for adolescents which should in turn improve patient outcome.

### Sample size

Qualitative data was collected to explore the health needs of ALPH. The key stakeholders comprise a diverse group of experts (n = 30) and adolescents (n = 19). The experts were: 1) representatives from health care sector included pediatricians, clinicians, psychiatrists, psychologists, counselors, 2) representatives from non-governmental organizations (NGO) included social workers, public health researchers, 3) representatives from national health program, and 4) primary caregivers of adolescents. Of the 19 adolescents, 12 were adolescents living with perinatally acquired HIV (ALPH) and 07 were HIV uninfected and affected adolescents. Data from adolescent interviews have been used to support findings from stakeholders’ analysis.

### Participant recruitment and data collection

Purposive and convenience sampling techniques were utilized to identify the stakeholders from different geographical regions in India such as North (Delhi & Lucknow), South (Chennai & Puducherry), and West (Mumbai, Pune and Goa). The stakeholders were identified based on their experience in HIV management among children and adolescents, adolescent health issues and health programs. These experts from diverse fields were approached through emails/ phone calls and prior appointments were taken. The primary caregivers, and HIV infected and affected adolescents were identified from Antiretroviral Therapy (ART) Centers, government organizations, non-government organizations and community. Purpose of the study was explained to the potential participants and those who were willing, were recruited in the study. Eight HCPs either refused or did not respond after multiple contacts. Eventually after these multiple contacts these HCPs expressed their inability to provide information on the topic because they had not adequate experience of working with adolescents. Therefore, new respondents in the same category were approached to reach the data saturation. All the consenting participants were interviewed by the first author, a trained anthropologist for conducting qualitative research. The interview guides were focused on the health issues of adolescents, diverse needs of ALPH, barriers and facilitators in the management of ALPH. The interview guides were pilot tested prior to data collection. All interviews were conducted face to face in English and Hindi languages at the convenient and confidential location. All interviews were audio recorded. Field notes were taken and data were collected till theoretical saturation was achieved.

### Study limitation

Focus group discussions (FGDs) were not conducted because a very small number of HCPs had experience in the management of ALPH. We could not conduct FGD with ALPH because parents/ guardian were not willing to allow the ALPH for participation in a group. They had fear of breach of confidentiality of HIV status of adolescents. These were the limitations of this study.

### Data management and analysis

Data were transcribed verbatim and typed in Microsoft Word after being translated in English. Data were entered in qualitative software, QSR NUD*IST 6.0 for analysis. We used three step guidelines for coding the data [18]. The code list was developed both deductively from the interview guide and inductively using the principles of grounded theory [19]. Initially, five transcripts were read to break, examine and conceptualize the categories. Each category was read to bring in linkages between the categories emerging out of open coding. The emerging codes were again reviewed by SS and AV together. Similar codes were merged with consensus between both the authors. Finally, those core categories were selected which validated the relationships between categories. Subsequently, thematic analysis was performed.

### Ethics

The study was approved by Institutional Ethics Committee of ICMR-National AIDS Research Institute, Pune, India. Written informed consents were obtained from all the participants prior to interview and audio-recording. Written assents from minor participants and consents from their parent/ guardian were obtained.

## Results

### Participants’ profile

A total of 30 key stakeholders, between the ages of 32 and 65 years participated in this study. Among adolescent participants, the age group was 13–19 years. The profile of the participants is shown in Table 1.

**Table 1:** Profile of study participants.

Respondents’ category	Expertise	Experience (in years)	Geographical Location	n
Clinicians (Paediatricians and other clinicians)	HIV, Child & Adolescent health (Paediatric Centre of Excellence, Kalawati Sharan Children Hospital; Command Hospital; UNICEF, Private Adolescent health Clinic, YRG Care)	10–25	New Delhi, Lucknow, Chennai, and Mumbai	07
Adolescent Mental Health Expert (Psychiatrist/ Psychologist)	Adolescents’ mental health & HIV (IHBAS, AIIMS, SRMU, Sangath, Puducherry University)	15–30	New Delhi, Chennai, Goa, Puducherry	05
Program officials	HIV/ AIDS programs, children/ adolescents’ program (NACO, UNICEF, Plan India)	10–15	New Delhi, Lucknow	04
Social workers/ Public health researchers	Adolescents’ health issues, HIV counselling, community based studies (Udayan Care, YRG Care, ICRW, SRMU)	15–25	New Delhi, Chennai	04
Primary caregivers of adolescents	Parent (Mother/ father) or guardian of adolescent living with perinatal HIV	-	Lucknow and Pune	10
Adolescents	Adolescents who acquired perinatal HIV, adolescents not having HIV infection		Lucknow, Pune, and Delhi	19
**Total**				**49**

**Fig 1:**
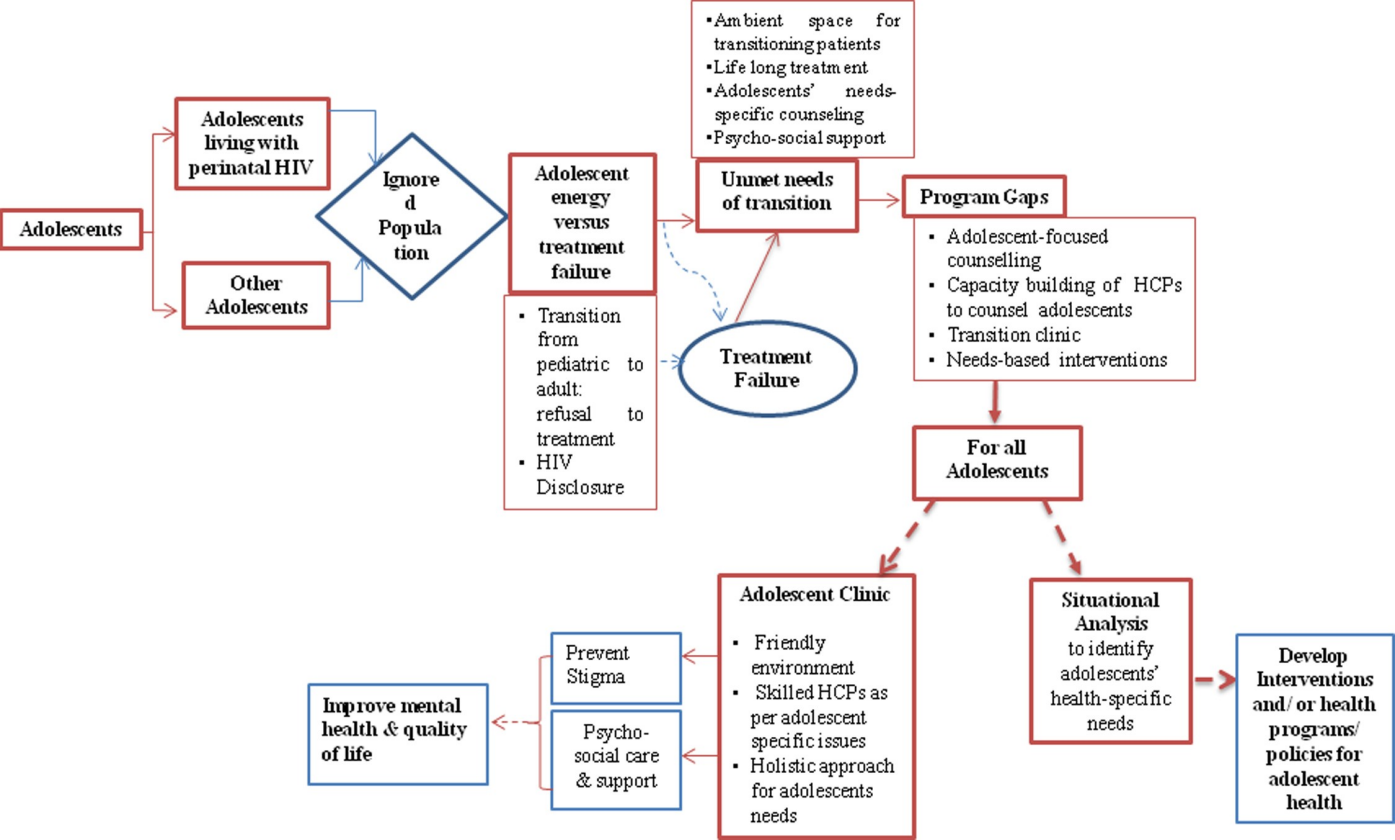
Analytical framework for emerging health needs of adolescents and gaps in health system for ALPH care.

Stakeholders felt that HIV/ AIDS among adolescents is a critical public health issue which requires a distinctive mandate. The analytical framework initially shows HIV infected and uninfected adolescents as two separate entities. However, the emergence of the theme namely, ‘lack of age segregated health data’ led to the theme of ‘ignored population’ which culminates into gaps in the program. These gaps were common to all adolescents who seemed to have unmet/ unaddressed needs of transition (Fig 1). ‘Stigma’ was an underlying theme from which the concept of common ‘adolescent clinics’ emerged. Under the ‘unmet/ unaddressed needs of transition’, need for 'adolescent clinic' emerged that underlined the Donabedian’s three tenets of care viz. structure, process and outcome; we also identified additional subthemes viz. ambient space, HCP-adolescent interaction, adolescent-specific counseling services, and psychosocial support including disclosure of HIV diagnosis for improving retention of ALPH in treatment and care. This additional finding of subthemes seemed likely to add innovation in addressing the needs of ALPH in India.

The results are discussed around the emerging themes of 1) Ignored population, 2) Unmet/ unaddressed needs of transition, 3) Treatment failures, and 4) Program gaps

### Ignored population

Prior to initiating discussion on the ‘ignored’ nature of this population, it would be prudent to discuss the situation of adolescent especially the ALPH. This forms the context and setting in which ALPHs are living. By virtue of being in the ‘adolescent age group’, this population not only remains ignored by the programmers but also because of stage of ‘adolescence’, these adolescents lose their usual independence for fun and recreation. Fear of stigma upon inadvertent disclosure by the child also makes parent vigilant towards their HIV infected child. A mother of ALPH brought out the concern of ‘adolescent stage’ as follows:

*"No*, *she [/participant's daughter/] does not go on school trips because they go to far-off places*, *I do not send her because there is no one to look after her*. *What if something happens between many boys-girls*? *Then what I will do*? *Hence I do not send her*.*"* [CG—08, Mother, Primary caregivers]

A guardian’s (grandmother) statement pertains to the control/ vigilance being exercised over the infected child.

*"[/cross talk/]… [Participant was talking about when her granddaughter goes to her friends' place] We send her brother with her [/HIV infected granddaughter/] [to her friend's place]*. *If her uncle is there at home*, *then we tell him to go and see*, *what = ALPH’s name = is doing [at friend's place]*. *She [/granddaughter/] is not allowed to go alone*.*"* [CG-04, Grandmother, Primary caregivers]

However, it is difficult for children to understand these reasons and they find this type of vigilance as unfair and therefore claustrophobic.

*“My mother does not allow to go with friends as she scared because ART is going on”* [AD-03, ALPH].

An adolescent questioned this behavior and seemed bewildered:

*"Nearby my house my friends live nicely*. *Means I am not*. *They enjoy*. *Now*, *few days ago there was a season of = Dandiya = [/a form of folk dance/]*. *When I asked my mother [/seek permission/]*, *she told me to go but when my friends came home to call me*, *then*, *my father refused permission*. *I also want to enjoy with them but it never happens*. *Other girls always tell me as to why I do not live like them*? *Why am I not enjoying so much*? *So I am unable to enjoy because of them [/parents/]"* [AD-26, ALPH]

All the stakeholders participating in this study pointed out that the adolescent population is missing the attention of the health programmers. They used following phrases to emphasize the need to bring in adolescent population as a separate entity in health care programs

*‘ignored’; ‘nobody was looking after those critical years of 10–20 or 10–18’ and ‘not looking at adolescent as a whole’*.

ALPH faced complex challenge of competing with their peers for government resources; there is no programmatic support especially for ALPH even though they emerge as vulnerable subset of this population. The PPTCT program official from UNICEF-NACO shared:

*“So far Government of India is not committed to give any kind of special treatment for these children [/ALPH/]*. *For HIV infected children*, *the social schemes are not there; so this is the problem*. *Only general schemes are there*.*”* [PO-04, Program Official]

A pediatrician described the binary of 1) paediatric and 2) adult care, subtly pointing towards the discomfort of pediatrician:

*"Adolescence was actually a period earlier which was pretty ignored*, *in the sense*, *adult physicians did not deal with them because they [/pediatricians/] felt that our job is over at 12 years [child]*. *When a child is undergoing such profound changes; nobody is comfortable dealing with them"* [PD-11, PCOE, KSCH, Clinician]

An ALPH carries dual burden of being at ‘adolescence’ and having ‘HIV infection’. The stigma at health care facility in a stage of life when they should be getting love and empathetic understanding, they are meted out stigmatizing treatment. An affected adolescent shares her anger over the stigmatizing behaviors that she observed at health care facility:

*"They [/doctor/] check other patients but not the one who is suffering from HIV*. *I get very angry* … *If you cannot touch them at least ask them what problems they have*. *They do not ask any questions*, *do not help*. *So what will that person do*? *He is unhappy with himself; this makes the person sadder in life*. *He might feel that there is no one for me*, *or to look after me*.*"* [AD-10, HIV affected adolescent]

An ALPH shares her own experience about the discriminatory behavior of some of the health care providers.

*"I had an operation*. *I had got [gall] stones*. *So during that operation doctor was delaying [the operation] means in November I got admitted… they stretched it up to December*. *After that once I had heard by mistake that she [/participant/] has HIV so… that is why there was delay*. *I had heard doctor’s and mother’s conversation by mistake*. *Then from outside*, *a doctor was called that such operation has to be done*. *Means the doctor who had to do [operation] was ready but his assistants were not*. *So that doctor and another doctor from outside did operation*.*"* [AD-01, ALPH]

A social worker calls for cognitive and empathetic understanding of adolescents:

*"We are just not a caring nation*, *we don’t have any value for children and children are the least thought about*. *We need to sensitize ourselves first*. *What are the needs of the children and why a child is doing what he is doing*? *To understand [/their/] non-verbal clues…"* [SW-06, NGO, Social Worker]

The adolescents who were generally given cocooned treatment since childhood felt very disturbed as they transitioned to adolescence and were meted different treatment.

*"I feel that why special treatment is there for this [/HIV/]*. *If it [/HIV/] is there then there*, *it is not our fault in that*, *so why so rudely* … *sometime doctors behave very rudely"* [AD-01, ALPH]

It was thus evident that needs of the ‘transition phase’ i.e. of adolescence is not getting addressed as this is an ignored marginalized population. Hence it would be critical to understand the needs of the adolescents and conduct gap analysis to identify the unmet or unaddressed needs of this population. The ensuing theme of ‘unmet/ unaddressed needs of transition’ emerged from the data and described as follows.

### Unmet/ Unaddressed needs of transition

It was noticed that respondents talked about all adolescents including ALPH for their health care needs. According to them, adolescents’ health care needs are unique and challenging by virtue of this particular stage of life itself. However, our data clearly shows the unmet need of ALPH for transition from pediatric care to adult care setting. Pediatric care has a protective environment; hence ALPH are least prepared for this transition and get often traumatized in the adult health care setting:

*“Once they are 18*, *they go to the adult [OPD]*, *then it becomes very difficult*. *Suddenly they are in an adult OPD on their own*. *In pediatric OPD*, *they are all cocooned*, *nurtured and suddenly in the adult OPD on their own- they are waiting in the queue*. *People do not have time for them so that transition becomes little difficult for them”* [PD-20, Pediatric HIV OPD, BJWMC, Clinician]

The transition experience of the ALPH was a contrast to the protective environment that they received during pediatric care. Adolescence itself is a stage where there is a need for investing quality time. Adolescence with HIV would require more of the quality time. An ALPH shared:

*"They [/counselor or doctor/] do not have time that is one reason and even not me… I cannot talk with anyone openly too early*.*"* [AD-01, ALPH]

An NGO representative shared example of life cycle approach of care for smooth transitioning from pediatric to adult care:

*“Our clinic is uniquely placed in such a way that both the pediatric clinic*, *adolescent clinic and adult clinic are in the same place*, *you know all same clinicians [/familiar clinicians/] take care of that*. *But we faced this [/transition problem/] from other centers where they [/ALPH/] have been taken care by pediatricians and when they are being transitioned to the adult physicians*, *these physicians do not have enough background information about these children*. *So I think that this type of link is important in any care system”* [MO-15, NGO, Clinician]

ALPH have more specific counseling needs than that available in standard adult HIV counseling set up. These needs do not get addressed in the current setting of government ART centers that are mainly positioned for adults:

*“Our counsellor cannot spend much time because of their busy schedule at ART center or the staff nurse cannot make concentrated efforts for these adolescents*. *The doctor and counsellor in the ART center are not able to manage the counselling required by these adolescents”* [PD-09, PCOE, KSCH, Clinician].

*"How is that at = [Hospital name] = *, *to talk with doctor the patient do not have privacy there* … *queue for ration [/subsidized food/] like that there is patients' queue at center*. *Not at all get privacy*, *such crowd and chit chat* …*means*, *how it happens*. *Door is open and 4-5people come inside in a queue means line starts from where only you are sitting [with HCP]*. *In crowd that child's concentration is not there to tell the doctor*.*"* [AD-31, ALPH]

For a satisfying outcome, it is evident the process of creating conducive health care environment to address the specific needs of the transition phase is crucial and essential. The sub-theme ‘ambient spaces’ reflects this processual issue as follows.

#### Ambient spaces

The current ART centers were not acceptable as appropriate ambient space for the ALPH. Adolescent do not like to transition to other clinics and it is very traumatic for them to leave their parent facility.

*"* …*the atmosphere there is different at clinic* …*people are lying over there*. *I used to feel very strange after going there*. *My transfer order was taking place there*. *But I said*, *no*. *Because I was habitual to go here… I started liking over here and I made people close to me over here*. *I feel nice talking with people over here and because of them I eat the tablets*. *I meet people*, *they do counseling*, *they convince very nicely so that I eat that tablets*. *The doctor was telling that you have to go at another center [/ART center/]*. *Since 5–6 year I am here*, *taking treatment* … *then I felt to cry*. *I cried a lot… very much*. *I cannot tolerate this thing*. *Whether it is near or far*, *I do not care*. *I just worry that I need to go away from people over here"* [AD-11, ALPH]

During transition phase, the adolescent requires a re-evaluation of their relationship to the external world, to the social world, and to one's own internal, psychic world. Therefore, a need to link with the external world merits serious and sincere attention. The link especially in case of ALPH is the counsellor as discussed below:

*“They need somebody to talk to them*. *So they cannot just have an ART Centre where they are given [ART]*. *There has to be somebody to talk to them*, *understand their needs*, *somebody whom they feel that they can come anytime and say whatever is going on*, *not their parents not their friends*. *So that kind of an atmosphere has to be there*.*”* [PD-20, Pediatric HIV OPD, BJWMC, Clinician]

*"* …*if anyone has [HIV] infection then that person has more need of a doctor and a good consult [/counselor/] means if any person cannot tell in the family but he can tell to doctor and if doctor tells him nicely or done his consulting [/counseling/] properly then his life can become good*. *For example*, *as doctor did counseling of me and said*, *‘nothing is there*, *only have to take 2 tablets in the life and live like normal person’*, *then I became stable*.*"* [AD-11, ALPH]

Stakeholders also discussed the structures required for the processes of counseling and for addressing specific needs of adolescents. Since everyone cautioned against adult health care setting, it was critical to identify the ‘ambient spaces’. Schools were suggested space for the much needed psycho-social support:

*“I do not think adolescents should come*, *especially to primary health centers*. *I think the ideal health care settings are schools where adolescents go to school*. *So counsellors in schools*, *peer-based counselling* …*”* [AP-19, NGO, Adolescent Mental Health Expert]

*"* …*for counsellors there should be specific cabin*. *If someone is standing behind their back then no one will talk*. *Rather than it is better to talk that much only which can be discussed there*.*"* [AD-12, ALPH]

Stakeholders advised against ‘Adolescent **HIV** Clinic’ to prevent any stigma against adolescents accessing these clinics. It was suggested that for the adolescents, a need-based guidelines for counselling should be developed. However, specific counselling for ALPH is critical because the very nature of ‘adolescence’ make them obstinate. It becomes difficult to make them adherent to treatment and hence there are negative outcomes such as stress, violence and treatment failures. ‘Treatment failure’ was another emerging theme which could be easily addressed if the focus for management is upon ‘adolescence’. The theme is discussed as follows.

### Treatment failures

As children transition from childhood to adolescence, they are known to become independent and their capacity to rationalize emerges. They take decision with their own logic and it becomes challenging in case of adolescents with chronic illness. Therefore, tendency for obstinate and tenacious behaviors rises among adolescents.

*"Sometimes get bored of this schedule [/daily medicine/]*. *Sometimes I came from outside… I leave from the class at 8*:*30*, *reach home by 9 and at 9 o’clock I have to have my medicine*. *So sometimes I feel like let it be*. *So sometimes I do not take [/medicine/]* … *At times when I have to be with friends or with people outside it is difficult and problems arise*. *So maybe that problem should not arise*.*"* [AD-12, ALPH]

*"Get angry*, *irritated that why I have started this [/ART/]*. *Sometimes I tell to mother also that I am not taking it [/tablet/] today*, *today is my exam I am not taking*.*"* [AD-01, ALPH]

Biologically, adolescence is an ‘energetic’ phase of life. The bounce and energy felt by adolescence leads to their obstinate views about their health. Our study identified the subtheme namely, ‘adolescent energy’.

#### Adolescent energy

We observed that respondents felt that adolescents generally are energetic and adept at having their own way especially if they did not want to do something. The sub-theme ‘adolescent energy’ pertains to this component of adolescent behavior. Understanding the adolescent energy and exuberance makes one understand the rash steps they take and rationalize them as correct. For example, refusal to be adherent to ART is easily rationalized by the biological capacity which made him feel healthy. The stakeholder quoted one of his adolescent patients:

"*I will not take [medicine]*, *I am absolutely fine*. *I can run a lot*! *[I] can do so much* [activities]’. *So then they [/ALPH/] start missing doses"* [PD-09, PCOE, KSCH, Clinician]

*“I always insisted about knowing it [/about diagnosis/]*, *I was adamant not to take medicines*. *I said*, *“I will throw the medicines away I do not want to take the medicines”*. [AD-03, ALPH]

*“I think it is good to tell them [about diagnosis] then they [/ALPH/] will take pills [/ART/] properly*. *If not tell them then they do not take pills regularly”* [AD-24, ALPH]

Thus, the treatment failures are quite evident among ALPH

*“Treatment failure occurs mostly in adolescents because they resist taking that [/ART/]”* [PD-09, PCOE, KSCH, Clinician]

Another emerging subtheme was related to health system and parents. HIV disclosure was the sub-theme that partially explains non-adherence and subsequent treatment failure among this population:

#### HIV disclosure

Protection and vigilance were the cited reasons by most of the parents for non-disclosure. They also feared losing respect of their child. Respondents shared that fear of innocent disclosure by adolescents and concerns regarding negative impact on their mental health also existed among the parents.

*“…the first major issue that we face with these adolescents is of disclosure”* [MO-15, NGO, Clinician]

*“*… *most parents do not disclose the HIV status* … *the biggest problem is the disclosure"* [PD-09, PCOE, KSCH, Clinician]

Therefore, the age defining exuberance is not the only reason for non-adherence but also a very objective management process of HIV disclosure can lead to non-adherence. Having no awareness of one’s own health status would be detrimental for any treatment adherence. The challenge of HIV disclosure not only pertains to stigmatizing nature of the disease but it also touches upon parent-child relationship. Despite the fears and inhibitions associated with disclosure among parents, all respondents emphasized that disclosure should be handled by health care providers (HCPs) along with parents/ guardians.

### Program gaps and care

It was important to understand what exists in the program for adolescents including ALPH. According to a child survival consultant [PD-05], adolescents have various needs pertaining to developmental changes and nutrition and they also want explanation about their treatment and drug side-effects. Their future aspirations reinforce the need for adolescent-focused counseling. However, counselling emerges as a gap:

*“Basically in India*, *what we see is that the counselling is something that is not being properly done in the facilities and that is why there is a sort of fear about their [/adolescents’/] illness*. *So they need to have proper counselling”* [PD-05, UNICEF, Clinician]

A consultant from an international agency shared her observations of counselling in a specific adolescent clinic (one state in India has piloted the concept):

*“Currently*, *we have a concept of adolescent health clinics in government system and the staff that is placed is basically to address the issues of reproductive health in adolescents*, *nutrition and to give some sort of psycho-social support*.*…I have been to a few counselling sessions at these adolescent clinics but I do not see them [/counsellors/] talking about the sexual health*, *high risk behavior*, *psycho-social needs vis-a-vis their hormonal changes"* [PD-05, UNICEF, Clinician].

#### Adolescent-focused counseling

Exploration of transition and specific needs of the transition stage revealed some program gaps which includes lack of special needs counseling centers within the health provider system, non-acceptance of facts on the part of the guardians, lack of self-confidence, and social stigma in general.

*“This is a group which is neither a child nor an adult*, *it is an in-between group and we really do not have a counselling thing which understands this group*. *So that is one that is required”* [PD-20, Pediatric HIV OPD, BJWMC, Clinician]

Elements of ALPH counseling were suggested:

*Protection from blaming oneself*, *no question on source of infection; build self-confidence despite HIV*, *ensure physical fitness-nutrition*, *taking care of oneself; being respectful for others & responsible behaviors for protection; knowing one’s rights to access resource and become advocates* [SW-12, ICRW, Social Worker].

#### Adolescent clinic

An ART program officer emphasized that there is need for adolescent focused clinics:

*"We do not have a concept of adolescent clinics in our country*. *We do require adolescent clinics*. *It may not be specifically for HIV but we require adolescent clinic where all issues related to adolescence whether they are HIV positive or HIV negative*, *are addressed”* [PO-08, NACO, Program Official]

#### Needs based interventions

Value of empathetic understanding of adolescents’ need was echoed by the programmers. According to them, the needs specific to adolescence, hitherto unknown, should be identified before planning any intervention for this age group. The national program official stated:

*“We need to identify what are the problems [of adolescents] and then think of addressing them and the ways to address them*. *It is not a one-point solution that what should we have*. *Basically first we should identify the issues"* [PO-08, NACO, Program Official]

#### Collaborative approach of care

Emphasis was laid on focusing both on socio-cultural stage and biological stage of adolescence which could be successful if interdepartmental collaborative horizontal programs are developed:

*“It is about the children*. *So*, *you have to involve all departments then only you will get the results*. *Not one single department can handle it*. *They have to involve social department; they have to involve ICDS [Integrated Child Development scheme] also; they have to involve this for what we call as special schemes for HIV [infected] children*. *It should not be a vertical program*, *you have to go with the horizontal [program] and have to merge with other departments also”* [PO-04, UNICEF-NACO, Program Official]

Forming collaboration was echoed. Linkages with psychologist, pediatricians and adult physicians was suggested:

*“They need a lot of psychological support*, *they need good psychotherapist and psychologists who should support*, *who should be good listeners*. *We should be linked to this psychiatric department or a child psychologist should come and talk to them [/adolescent patients/] because you need at least half an hour to 45 minutes’ session to talk to these children”* [PD-09, PCOE, KSCH, Clinician]

*“Definitely there should be collaboration between the pediatricians and adult clinicians so that link and continuation is maintained*. *I think that should not be excuse*. *You have to develop a strategy where appropriate counseling*, *appropriate package of care should be implemented for everybody”* [MO-15, NGO, Clinician]

## Discussion

The success of PPTCT program in India, calls for program and policy response to ensure clinical, social and structural management of ALPH’s specific needs. Lack of age disaggregated data on health in the country is a critical gap and it poses barrier to formulate adolescent/ ALPH focused programs and policies. Other studies have also indicated that interventions evaluated to date have not been tailored to the needs of adolescent age group [10]. A study conducted in United States reported several barriers to ALPH’s successful linkage to care viz. providers lacked ‘friendliness’, and adolescents’ developmental stage made managing their illness challenging [20]. Similar barriers emerged in our study also, thus reinforcing the fact that there is paucity of policies pertaining to adolescent management and care. The study participants emphasized that the rapidly shifting meanings attached to the transition to adulthood was crucial for management of adolescents as reported by others also [21]. ALPH were reported to face additional transitions than an average adolescent. In our study, these additional transitional events were manifested in hyper-vigilance by the adults, control over socializing and out-door activities and constant vigilance on hiding HIV status. The study brings out health related transitions as dual burden for ALPH in terms of prohibitions related to adolescent reproductive and sexual health and gender relationship.

Regarding skills and capacity, respondents reported lack of capacity to deal with adolescent health issues. This is similar to studies reported from Zimbabwe, Africa which suggests that primary HCPs often do not recognize HIV infection among adolescents [22]. In this study, pediatricians reported being uncomfortable with adolescents even if they had managed them as children. Low confidence in dealing with adolescent issues surfaced repeatedly. It seems that the real problem lies with the health system which requires either pediatric or adult skills while it is evident that adolescents do not embody any of the two- adult and pediatric spaces [23,24]. South Asia, especially India has one of the largest adolescent populations in the world [25,26]. Capacity building among HCPs to manage adolescents’ health, especially ALPH health therefore, emerges as an imperative for health programmers.

The objectivity of age oriented but not the age segregated health system policy is debatable. An ALPH transitions suddenly to the adult care system which lacks the understanding towards their changing biological and psychosocial needs. In Indian setting, there is no guideline for this transition, hence HCPs reported treatment interruptions and sometimes treatment failure among ALPH. Studies have shown that vertically infected ALPH are at higher risk of treatment failure and drug resistance due to susceptibility to side-effects because of long term drug usage and, sensitivity to physical body changes, growth and nutrition which complicates their ART management [27]. Adolescent energy and rationalization of their risky behaviors and practices could be the crucial indicators of impending treatment interruptions.

Stakeholders discussed a novel idea of using life cycle approach suggesting pediatrician and adult physician to deal with adolescent issues during treatment. The New York State Department of Health AIDS Institute guidelines also recommends placing adult care provider in pediatric settings [28]. We recommend to establish a preparatory ‘familiarity or pre-adult’ process which prepares an ALPH for adult health care setting. The age of preparatory phase could be nearer, at least one year prior, to the attainment of adult legal age. Accordingly, this strategy may help in understanding and preparing reports upon developmental and behavioral issues of ALPH which should be passed from ‘familiarity or pre-adult’ HCP to HCP in adult health care setting. Capacity building of HCPs to manage adolescents’ health issues is recommended. In the absence of no health policy for adolescent health in India, situational analysis to identify and shape health priorities of adolescents is also recommended.

Children are expected to be on anti-retroviral therapy for 20 years longer than an adult [29] and the socially embedded reasons for non-adherence show that investment in biomedical interventions alone will be inadequate [30]. Providing the psycho-social support is critical for management of ALPH. The adolescent counselling package should include addressing developmental issues, life skills, issues of guilt, shame and blame, nutritional and physical fitness needs, disease management and treatment related needs. Rights based approach has also been recommended.

In response to the specific needs of adolescence, establishment of ‘adolescent transition clinic’ would be a step forward towards Joint United Nations Program on HIV/AIDS 90–90–90 targets [31]. HIV testing and linkage to treatment, sustaining viral suppression [32] or increase in loss to follow-ups among children rolling over to adult care has been a challenge world-wide [33,34]. The adolescent transition clinic would be critical to mitigate loss to follow-up among ALPH. This should be prioritized by the programmers on an urgent basis as 21% of total population in India is that of adolescents [35,36]. Studies have shown that adolescent specific space allows for more privacy, delivery of comprehensive services by trained staff to meet the developmental needs of adolescents which increases the adolescents’ participation in their own care [35,37]. Although, ALPH require a complex healthcare system that engages with supportive well trained staff and adolescent friendly physical and social environment; all stakeholders unanimously cautioned against ‘adolescent HIV clinic’ to prevent inadvertent disclosure and stigma. A holistic approach should be adopted where adolescents can receive all services including mental health care and psycho-social support under one roof.

Access to health services among adolescents has been cited as barrier. Beneficiary students of Adolescent Education Program gave generally positive feedback and found this as an opportunity to explore sexual matters in a safe space [38]. Familiarity, neutrality and least threatening spaces for ALPH is required and schools fulfill these needs amply. Schools are recommended as settings for easy access among this population. We recommend to establish ‘adolescent transition clinics’ as an integral part of health and education system where common issues of adolescents, both HIV infected and uninfected, can be addressed.

Involvement of parents/ guardians could improve the utilization of health services by adolescents. A study from India reports poor parental relationship which is associated with early sex initiation [39]. However, respondents recommended parents’ engagement during HIV disclosure. To prevent any harm, relationship assessment needs to be part of the program prior to parental engagement. Interventions for relationship building needs to be designed and tested.

Multi-sectoral approach should be adopted by including education sector, departments like social welfare, ICDS and non-governmental organizations. Integration of care services and linkages can improve care and reduce missed opportunities for adherence support as integration of care is an important strategy to improve patient retention in long-term HIV care and treatment [40,41]. Adolescent HIV/AIDS in India demands innovation in the provision of adolescent HIV-related care. Limited research related to quality of care for ALPH in India necessitates the research in this area to generate evidences that will be utilized for improving the services for ALPH.

## Conclusions

“Transition clinics” to meet the health needs of adolescents is the need of the hour. School can be one of the settings for adolescent health care. Adolescent-specific counseling was an expectation from program. Capacity building of HCPs for management of adolescent health was a felt need among stakeholders in India. Special programs and resources for ALPH is recommended. Age-disaggregated data needs to be urgently obtained to inform/tailor programs and policies. Subsequently, situational analysis should be done to identify and shape health priorities of adolescents. Programs and policies should be informed by the adolescents and youth they are intended to serve.

## Supporting information

S1 Table COREQ checklist. (DOC)
