# Supplementary material for: Healthcare needs and programmatic gaps in transition from pediatric to adult care of vertically transmitted HIV infected adolescents in India
Source: PLoS One. 2019 Oct 29;14(10):e0224490. doi: 10.1371/journal.pone.0224490 (PMC6818794; doi:10.1371/journal.pone.0224490)
Supplement: S1 Table — (DOC) [file pone.0224490.s001.doc]

**Manuscript:** Healthcare needs and programmatic gaps in transition from pediatric to adult care of vertically transmitted HIV infected adolescents in India.

**Consolidated criteria for reporting qualitative research (COREQ) checklist**

| **Item No.** | **Topic** | | **Guide questions/description** | **Reported on Page No.** |
| --- | --- | --- | --- | --- |
| **Domain 1: Research team and reﬂexivity** | | | | |
|  | *Personal Characteristics* | | | |
|  | Inter viewer/facilitator | Which author/s conducted the interview or focus group? | | Line nos. 93-94; Page 5#, Section: Methods |
|  | Credentials | What were the researcher’s credentials? E.g. PhD, MD | | Line nos. 93-94; Page 5#, Section: Methods |
|  | Occupation | What was their occupation at the time of the study? | | Line nos. 93-94;Page 5#, Section: Methods |
|  | Gender | Was the researcher male or female? | | Female |
|  | Experience and training | What experience or training did the researcher have? | | Line nos. 93-94; Page 5#, Section: Methods |
|  | *Relationship with participants* | | | |
|  | Relationship established | Was a relationship established prior to study commencement? | | Line nos. 86-92; Page 5#, Section: Methods |
|  | Participant knowledge of the interviewer | What did the participants know about the researcher? e.g. personal goals, reasons for doing the research | | Line nos. 91-92; Page 5#, Section: Methods |
|  | Interviewer characteristics | What characteristics were reported about the interviewer/facilitator? e.g. Bias, assumptions, reasons and interests in the research topic | | Line nos. 93-94; Page 5#, Section: Methods |
| **Domain 2: study design** | | | | |
|  | *Theoretical framework* | | | |
|  | Methodological orientation and Theory | What methodological orientation was stated to underpin the study? e.g. grounded theory, discourse analysis, ethnography, phenomenology, content analysis | | Line nos. 104 - 111; Page 6#, Section: Methods, Subsection: Data management and analysis |
|  | *Participant selection* | | | |
|  | Sampling | How were participants selected? e.g. purposive, convenience, consecutive, snowball | | Line nos. 85-86; Page 5#, Section: Methods |
|  | Method of approach | How were participants approached? e.g. face-to-face, telephone, mail, email | | Line nos. 84-91; Page 5#, Section: Methods |
|  | Sample size | How many participants were in the study? | | Line 80-82; Page 4 - 5#, Section: Methods |
|  | Non-participation | How many people refused to participate or dropped out? Reasons? | | Line nos. 92-93; Page 5#, Section: Methods |
|  | *Setting* | | | |
|  | Setting of data collection | Where was the data collected? e.g. home, clinic, workplace | | Line nos. 96-98; Page 5#, Section: Methods |
|  | Presence of non-participants | Was anyone else present besides the participants and researchers? | | No, Line nos. 96-98; Page 5#, Section: Methods |
|  | Description of sample | What are the important characteristics of the sample? e.g. demographic data, date | | Page 6-7#, Section: Results; Table 1 |
|  | *Data collection* | | | |
|  | Interview guide | Were questions, prompts, guides provided by the authors? Was it pilot tested? | | Line nos. 94-96; Page 5#, Section: Methods |
|  | Repeat interviews | Were repeat interviews carried out? If yes, how many? | | No |
|  | Audio/visual recording | Did the research use audio or visual recording to collect the data? | | Line no. 98; Page 5#, Section: Methods |
|  | Field notes | Were ﬁeld notes made during and/or after the inter view or focus group? | | Line nos. 98- 99; Page 5#, Section: Methods |
|  | Duration | What was the duration of the inter views or focus group? | | 30-80 minutes |
|  | Data saturation | Was data saturation discussed? | | Line nos. 98-99; Page 5#, Section: Methods |
|  | Transcripts returned | Were transcripts returned to participants for comment and/or correction? | | No |
| **Domain 3: analysis and ﬁndings** | | | | |
|  | *Data analysis* | | | |
|  | Number of data coders | | How many data coders coded the data? | Line nos. 108-109; Page 5-6#, Subsection: Data management and analysis |
|  | Description of the coding tree | | Did authors provide a description of the coding tree? | Line nos. 105-111, 120-132; Page 6,7#; Subsection: Data management and analysis; Section: Results |
|  | Derivation of themes | | Were themes identiﬁed in advance or derived from the data? | Line nos. 105-111; Page 6#, Subsection: Data management and analysis |
|  | Software | | What software, if applicable, was used to manage the data? | Line no.104; Page 6#, Subsection: Data management and analysis |
|  | Participant checking | | Did participants provide feedback on the ﬁndings? | No |
|  | *Reporting* | | | |
|  | Quotations presented | | Were participant quotations presented to illustrate the themes/ﬁndings? Was each quotation identiﬁed? e.g. participant number | Line nos. 146-334; Page 8 to 17#, Section: Results |
|  | Data and ﬁndings consistent | | Was there consistency between the data presented and the ﬁndings? | Yes |
|  | Clarity of major themes | | Were major themes clearly presented in the ﬁndings? | Yes, Line 135-334; Page 8 to 17#, Section: Results |
|  | Clarity of minor themes | | Is there a description of diverse cases or discussion of minor themes? | Discussion of sub/minor themes  Page 8 to 17# |

Developed from:

Tong A, Sainsbury P, Craig J. Consolidated criteria for reporting qualitative research (COREQ): a 32-item checklist for interviews and focus groups. *International Journal for Quality in Health Care*. 2007. Volume 19, Number 6: pp. 349 – 357
